# Issues in Child and Adolescent Inpatient Assessment and Evaluation After Discharge: Protocol for App Development and a Randomized Controlled Trial

**DOI:** 10.2196/10121

**Published:** 2018-11-12

**Authors:** Kristian Hansson, Björn Axel Johansson, Claes Andersson, Maria Rastam, Sophia Eberhard

**Affiliations:** 1 Department of Child and Adolescent Psychiatry Regional Inpatient Care, Emergency Unit Skåne University Hospital Malmö Sweden; 2 Division of Child and Adolescent Psychiatry Department of Clinical Sciences Lund Lund University Lund Sweden; 3 Department of Criminology Faculty of Health and Society Malmö University Malmö Sweden

**Keywords:** app, data collection, mobile phone, research protocol, screening

## Abstract

**Background:**

New methods are needed for collecting data of in- and outpatients and for improving outpatient compliance after discharge. Mobile technologies, such as smartphone apps, have shown promising results, (eg, helping unwell people by offering support and resources). Screening for the condition, including comorbidities, is a vital part of psychiatric care. Comorbid conditions, especially in emergency evaluation, are often missed, leading to inaccurate diagnosis and treatment. One way of improving diagnostic accuracy is to use a structured diagnostic process. Digitalized screening and follow-up have the advantage of making administration and scoring easier and less time consuming, thereby increasing response rate. To address these problems, we decided to create a smartphone app called The Blue App. The Blue App was developed through 6 steps, described in the manuscript.

**Objective:**

The aim of this paper is to describe (1) the development of The Blue App and (2) 2 planned research studies to evaluate the app.

**Methods:**

Two studies will be performed. Study 1 has a descriptive design, mapping comorbidities before and after the introduction of The Blue App. Study 2 has a randomized controlled design, measuring compliance with outpatient treatments as well as depressive symptoms, rated as changes in Montgomery-Åsberg Depression Scale scores during a 1-year follow-up.

**Results:**

We have described app development. Data collection for Study 1 started in autumn 2017. Study 2 will start in autumn 2018. We expect to have enrolled the 150 patients in Study 2 by December 2019. Final results will be published in a scientific journal.

**Conclusions:**

A technically advanced and easy-to-use Web-based mobile phone app corresponding to the unit’s needs was developed, and 2 studies are planned to evaluate its usefulness.

**International Registered Report Identifier (IRRID):**

RR1-10.2196/10121

## Introduction

Emergency units with 24/7 evaluation and admission facilities are important services for stabilizing adolescents with mental health crisis [1,2]. On admission to a unit, patients often need extensive mental health resources [3]. Demand for health production is high in emergency units when inpatient stays are short [4].

The largest intervention effect is reached during the first week of admission [5]. Short inpatient stays for acute stabilization are a challenge when it comes to structured diagnostic screening. Despite the availability of validated diagnostic screening instruments, pen-and-paper versions lead to poor response rates and consume resources, making their use less attractive [6]. The current toolbox is outdated for use in our population of adolescents, and new methods are needed for inpatient data collection and for improving outpatient compliance. Mobile technologies, such as smartphone apps, have shown promising results in terms of data collection and as an intervention for behavioral change, but little empirical evidence has been found to date [7,8].

Literature in the field of child and adolescent psychiatric inpatient emergency care is scarce, and studies are limited to characteristics of admitted patients [3]. Therefore, little is known about the course of illness after discharge. In Sweden, there has been no comprehensive evaluation and follow-up of children and adolescents admitted and discharged from psychiatric emergency units.

Diagnosing the condition and screening for comorbidity using validated instruments is a vital part of psychiatric care. Moreover, a structured screening procedure is also perceived as positive and valid by patients and their parents [9].

In psychiatric care, diagnostic interrater reliability among clinicians is weak [10]. Comorbid conditions are commonly missed during psychiatric evaluations, leading to inaccurate diagnosis and treatment. One way of increasing diagnostic accuracy is to use a structured diagnostic process, involving diagnostic screening instruments [11].

Digitalized screening and follow-up make administration and scoring easier and less time consuming for patients, parents, and staff. This helps increase the frequency of completed systematic psychiatric screenings. It also has the advantage of availability, as questionnaires can be sent to and answered on patients’ smartphones, increasing response rates [6]. This structured data collection would enable studies of child and adolescent psychiatric emergency inpatients in further research.

Apps have proved effective in helping unwell people, by offering support, resources, and information [7]. In recent years, several new smartphone-based solutions have been developed for mental health in general and specifically for child and adolescent psychiatry [12-16]. However, apps developed by health care professionals for adolescents must be innovative, useful, and fun to compete with apps not encouraging healthy behaviors [7].

To address these problems, we decided to create a smartphone app: The Blue App (referring to blue as the color of hope, as well as expressing moodiness). The Blue App is a Web-based tool made to resemble and function as a mobile app.

The aim of this paper is to describe the development of The Blue App and to present 2 research protocols aimed at evaluating the app.

## Methods

### Development of The Blue App

The process of developing The Blue App, from concept to working product, was divided into 6 steps.

#### Step 1. Identifying the Need for Quick and Easy Information Gathering

Since 2010, members of our group have introduced and developed interactive voice response (IVR) into Swedish child and adolescent psychiatry. This is a technology in which a server is programmed to use scripts in interaction with the user on their cell phone [9,14]. Johansson et al [17] have shown that emergency patients had their own cell phones and that the response rate among participating patients (N=60) was promising as each individual responded on average 91% (365/402) of their calls. An answering frequency of 100% was shown by 71% (30/42).

Our work with IVR inspired us to further develop technology to meet the unit’s needs regarding improved methods for the diagnostic and follow-up processes. In 2013, we published our first IVR paper [17]. Simultaneously, we were discussing the shortcomings at the unit regarding the identification of comorbidities. We were also discussing the development of a more sophisticated follow-up tool that would provide feedback using a visual presentation of changes in symptoms over time. At the same time, smartphones were becoming more common among adolescents. This enabled us to integrate our previous work with our efforts to widen the use of screening questionnaires in a better package than the older cell phones could offer.

To address the issues related to short inpatient stays and need for improved diagnostic screening, we decided to create a smartphone app based on our previous work.

#### Step 2. Identifying Desired Information and Choosing Adequate Screening Questionnaires

In addition to clinical evaluations, we wanted to screen for and describe the psychiatric conditions that were most frequently presented at emergency units. The most common reasons for admission to the unit were suicidal ideation, severely depressed mood, and acute crises. Our experience corresponds well with literature on child and adolescent psychiatry with regard to frequent comorbid states [2]. We also wanted to map changes in symptom severity before and after admission.

The validated screening questionnaires were chosen in collaboration with national key opinion leaders and with regard to frequent comorbid states in child and adolescent psychiatry [18-20].

The following 10 questionnaires were chosen:

1. Montgomery-Åsberg Depression Scale (MADRS-S): a 9-item diagnostic self-rating questionnaire measuring depressive symptoms during the previous 3 days [21]
2. Mini International Neuropsychiatric Interview for Children and Adolescents (MINI-KID): a semi structured diagnostic interview concerning the core psychiatric disorders among children and adolescents [22]
3. Alcohol Use Disorders Identification Test-Consumption: a 3-item screening questionnaire regarding alcohol use [23]
4. Drug Use Disorders Identification Test: an 11-item screening questionnaire regarding drug use [24]
5. Hopkins Symptom Checklist-10: a symptom inventory that measures signs of anxiety and depression [25]
6. Sheehan Disability Scale: a scale assessing functional impairment in domains of work or school, social, and family life [26]
7. Autism Spectrum Screening Questionnaire: a 27-item checklist that assesses symptoms characteristics of high-functioning autism spectrum disorder [27]
8. Swanson Nolan and Pelham Questionnaire: a 30-item questionnaire assessing attention-deficit/hyperactivity disorder symptoms and behavioral problems [28]
9. Deliberate Self-Harm Inventory-9: a scale showing the frequency of different ways of self-harming [29]
10. Treatment Satisfaction Scale 2: a 6-item measure of treatment effectiveness [30] (included to evaluate the satisfaction of treatment but not a part of the research protocols)

#### Step 3. Examining the Feasibility in an Emergency Psychiatric Unit: Pilot Study

To evaluate the feasibility of the screening procedure, a pilot study was performed with adolescents admitted to the psychiatric emergency unit. The first aim was to assess the feasibility of administration of 10 questionnaires in a small consecutive population. The second aim was to learn more about the clinical relevance of the systematic screening, that is, would the results indicate more comorbidities than found in the medical records? Patients admitted to the unit between February 11, 2014, and March 14, 2014, were invited to participate and complete the 10 questionnaires regarding psychiatric morbidity and comorbidity to be included in The Blue App. All screening questionnaires involved using pen and paper.

We included 16 patients (12 girls and 4 boys; age: mean 15.6 (SD 1.31) years). Of these, 12 completed all the pen-and-paper screening questionnaires, whereas 4 patients could not complete their screening procedures due to early discharge. Of 10, 9 diagnoses at discharge were confirmed using MINI-KID. MINI-KID also identified, on average, 5 diagnostic areas of potential psychiatric interest, including the hazardous use of alcohol or substance abuse, none of which had been clearly addressed during the inpatient stay or mentioned in the psychiatric records.

#### Step 4. Getting Organizational Acceptance: Feasibility Study

The next step was to secure a way forward for The Blue App. As our aim was to use the tool in daily clinical practice, we decided to apply for funding from the funding body for all public psychiatric health care in Scania, South Sweden. All potential new procedures in Swedish public health care require a thorough assessment procedure to clarify the cost-benefit ratio, safety, and potential risks (feasibility study). The aim of this feasibility study was to clarify the requirements of The Blue App and to elucidate the cost-benefit and safety aspects so as to generate a basis for decision making for the funding body. Another part of the feasibility study was to collect knowledge about alternative solutions allowing the same functionality by investigating if comparable products were available on the market.

The feasibility study included about 15 meetings with the IT and finance departments. Clinicians’ needs were highlighted, which resulted in a thorough description of the technical requirements for 90 user cases, including mock-ups, flowcharts, and wireframes. The study concluded that no existing solution was available on the market. The final document was presented to the funding board with the working title “The Blue App.” The board approved funding in May 2015.

#### Step 5. App Construction

After approval, the next step was to call for public procurement using the feasibility study documents. In total, 3 tenders were considered, and representatives from each company were interviewed. The clinicians working on The Blue App were involved in decision making. Work with the company chosen, Stretch Öresund, proceeded with workshop sessions, creating a shared language and translating clinicians’ needs to the mindset of app architects. The work continued with meetings being held every 2nd week for a 6-month period, during which mutual feedback on the progress of The Blue App was given. The company’s CEO met with the unit staff to demonstrate the app and collect feedback to increase the chance of successful implementation.

The work resulted in an app corresponding with the unit’s needs. The structure of the development process, with frequent and regular meetings, was necessary to avoid misunderstandings. The development process allowed the app architects to gain a profound understanding of psychiatric care and to adapt solutions to the unit’s requirements.

#### Step 6. Workshop With Adolescents: What Do the Patients Think?

During the development of The Blue App, feedback was obtained through a workshop with adolescent end users. The main aim was to assess the user-friendliness of the app. We arranged the workshop in spring 2016, during which 10 adolescents, 5 boys and 5 girls aged 14-17 years, participated. Of these, 2 adolescents were undergoing psychiatric outpatient treatment. The participating adolescents were introduced to the app and then navigated through The Blue App follow-up user interface. The participants found the app easy to navigate and suggested some minor improvements, such as the integration of graphic design.

### Planned Research Studies to Evaluate The Blue App: Research Studies 1 and 2

The objective was to design several studies to evaluate The Blue App. In the first study (Study 1), we aim to assess whether additional comorbid conditions could be identified using The Blue App. In the second study (Study 2), we want to evaluate whether the use of The Blue App can improve outpatient compliance and treatment outcome through feedback of psychiatric symptoms.

### Design

#### Study 1

In Study 1, we want to map the prevalence of psychiatric comorbidity found in the medical records before and after the introduction of The Blue App. The hypothesis is that more diagnoses will be found in the records after the introduction of The Blue App.

#### Study 2

**Figure 1:**
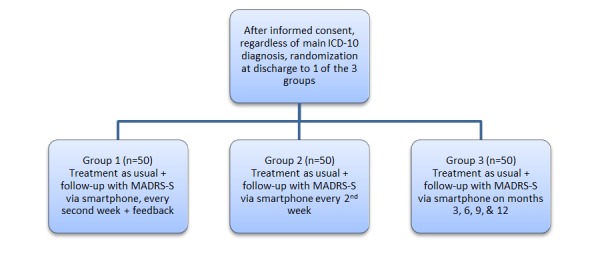
Study design with 3 randomization groups for the follow-up study at the Child and Adolescent Psychiatric Emergency unit, Malmö, Sweden. ICD-10: International Classification of Diseases, 10th revision; MADRS-S: Montgomery-Åsberg Depression Scale.

At discharge, eligible patients will be offered participation in a 1-year follow-up study using a randomized controlled design (Figure 1). After receiving informed consent, patients will be randomized into 3 groups and asked to answer questions regarding depressive symptoms (MADRS-S) at different time intervals over a period of 1 year after discharge. The time intervals were chosen to minimize dropout while being long enough to detect effects in the patient. We undertook a power calculation, as we found no comparable studies. Based on estimated scores on the depression rating scale (MADRS-S) at discharge, we expect scores at discharge to be around 30, with SD 7.1.

The null hypothesis for Study 2 is that no significant difference is found in the randomized controlled trial; that is, the feedback given through The Blue App would not improve the outcome or compliance. In the alternative hypothesis, we expect all patients to reduce their MARS-S scores during the study period and expect a difference of about 4 points on MADRS-S between intervention and control groups. With a power of .8, we will therefore need 3 groups of 50 patients each in order to detect differences at a 5% level.

#### Setting and Participants: Studies 1 and 2

Scania in southern Sweden has a population of over 1 million, of which 280,000 are children and adolescents. The university hospital in Malmö, the major city in the area, provides the only psychiatric emergency unit for children and adolescents, with 11 hospital beds. The hospital admits 350 unique patients annually together with a parent for acute stabilization. The most common reasons for admission are suicidal ideations, severely depressed moods, and acute crises. A majority of the patients are aged 13-17 years, with an even gender distribution.

A treatment plan is drawn up by the unit physician together with patients and parents. Nearly 15% of the patients are treated according to the Swedish Compulsory Mental Care Act. The unit team consists of physicians, psychologists, social workers, nurses, and treatment staff, often in collaboration with open-care units, schools, and social authorities. Before discharge, a rescue plan is drawn up together with patients and parents in the event of a recurrent emergency situation. Nearly 95% of the patients are discharged within a week of admission.

### Procedure

#### Study 1

All eligible patients will be offered participation in Study 1 and will be invited to complete the questionnaire (exclusion criteria: language other than Swedish, admission for <24 hours, severely ill, age <12 years old). We expect about 190 of the 350 patients admitted per year to be eligible for inclusion. The feasibility study indicated little attrition, but we anticipate that about 30% might not want to participate, leaving us with about 130 per year for possible inclusion.

**Figure 2:**
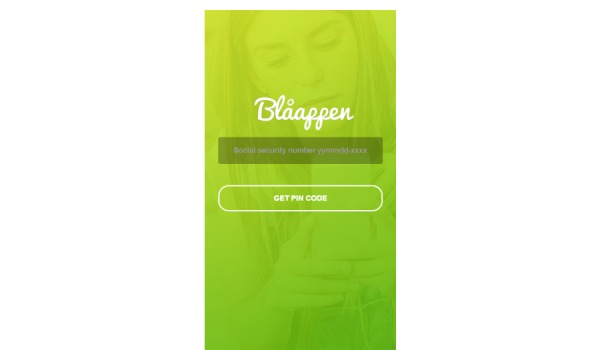
Screenshot of The Blue App login screen. Source: Region Skåne.

Patients and parents will be asked to complete questionnaires on psychiatric symptoms, treatment satisfaction, and quality of life. Included patients will be registered on The Blue App, allowing them to log in via their smartphone using a personal identity number at the log-in page (Figure 2).

The patients will then receive a short message service text message with a one-time password, enabling them to enter The Blue App and answer the questionnaires. It is also possible to generate one-time passwords and complete the questionnaires via The Blue App staff administration view. Parents can use their child’s log-in when answering the parental questionnaires.

**Figure 3:**
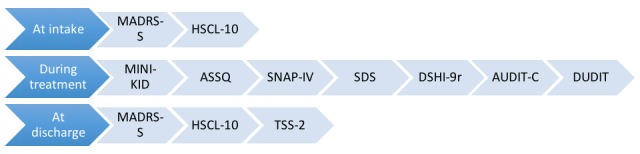
Questionnaires used at the different stages during the inpatient stay at the Child and Adolescent Psychiatric Emergency Unit, Malmö, Sweden. ASSQ: Autism Spectrum Screening Questionnaire; AUDIT-C: Alcohol Use Disorders Identification Test-Consumption; DSHI-9: Deliberate Self-Harm Inventory-9; DUDIT: Drug Use Disorders Identification Test; HSCL-10: Hopkins Symptom Checklist-10; MADRS-S: Montgomery-Åsberg Depression Scale; MINI-KID: Mini International Neuropsychiatric Interview for Children and Adolescents; SDS: Sheehan Disability Scale; SNAP-IV: Swanson Nolan and Pelham Questionnaire; TSS-2: Treatment Satisfaction Scale 2.

The screening process at the unit will be divided into 3 stages (Figure 3). The first stage is defined as the first 24 hours at the unit. The second stage, during treatment, is defined as the time between intake and discharge. The discharge stage is defined as the last 24 hours at the unit. Patients and their parents will be given access to the questionnaires at each stage, which they will complete with the help of staff. At the unit, the questionnaires will be administered mainly on tablets, but can also be filled in via the patients’ own devices.

#### Study 2

At discharge, participation in study 2 will be offered to all patients who complete the questionnaires for Study 1 (Figure 3).

**Figure 4:**
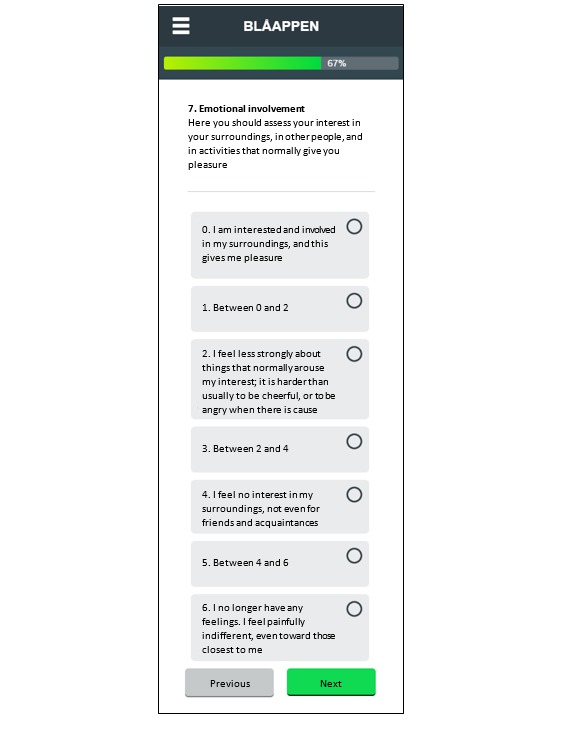
Screenshot of the MADRS-S (Montgomery-Åsberg Depression Scale) questionnaire item 7. Source: Region Skåne.

All patients in the study will receive treatment as usual at their outpatient units. Patients will use the same log-in procedure as in Study 1. With app-assisted technology, a link to the MADRS-S questionnaire (Figure 4) will be sent to all participants at predefined intervals depending on randomization.

After discharge, researchers and staff will not be able to trace the group to which a patient is assigned. Patients readmitted to the ward during follow-up will remain in the study.

**Figure 5:**
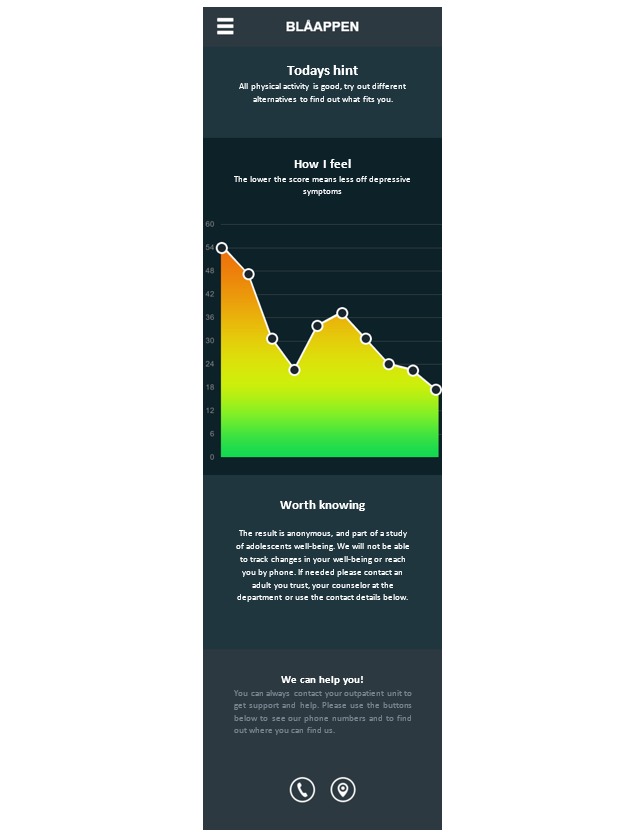
Screenshot of the MADRS-S (Montgomery-Åsberg Depression Scale) feedback screen for group 1. Source: Region Skåne.

In group 1, feedback on depressive symptoms will be presented in a graph, together with a brief recommendation [31] on each follow-up occasion (Figure 5). A text below the graph will describe how patients can contact the emergency unit if they experience very severe symptoms or danger, with a button to make a direct call to the 24/7 emergency facilities.

Dropout analysis will examine for any group differences in terms of gender, age, and main International Classification of Diseases, 10th revision diagnoses. Information regarding reasons for missed appointments and dropouts will be taken from the medical records. Outcome measures are changes in MADRS-S scores and treatment compliance, as measured by attended outpatient appointments during the study period.

### Ethical Approval

No compensation will be offered. Subjects in the study will be protected by the informed consent process. The Regional Ethical Board in Lund has approved the study (2013-06-19; Nr 423/2013).

### Statistical Analysis

The two studies will be analyzed separately, and baseline variables will be analyzed with descriptive statistics. Pearson’s chi-square tests will be used to determine differences between the groups in terms of gender distribution and proportion of participants scoring above cut-off in the questionnaires used.

In Study 1, we will use descriptive statistics (mean, SD, median, interquartile range, continued variables, frequency and counts, categorical variations, and exact CIs for proportions) to describe the cohort and analyze differences in the number of psychiatric diagnoses before and after the introduction (*t* test) of The Blue App [32].

In Study 2, we will analyze differences between the 3 groups in terms of changes in MADRS-S scores and treatment compliance. Outcome measures (MADRS-S scores and dropout from outpatient care) will be analyzed according to intention to treat. Linear mixed model analyses will be used to identify changes over time in depressive symptoms and to examine compliance with outpatient treatments. In these analyses, group, time, and the intercept between the groups and time will be used as fixed effects and subjects as random intercepts.

## Results

The app development is described. Data collection for Study 1 started in autumn 2017. Study 2 will be initiated in autumn 2018. We expect to have enrolled 150 patients for Study 2 by December 2019. Funding for both Study 1 and Study 2 have have been granted by Region Skåne, the regional council for health care in south of Sweden. Final results will be published in a scientific journal.

## Discussion

### Principal Findings

This study is a process description of a child and adolescent emergency inpatient unit, where an app was developed with the aim of identifying comorbidity and improving outpatient compliance and treatment outcomes.

### Strengths

The Blue App is a dynamic tool capable of adjustment to new demands, with variable settings, and to other units with potential to improve the transition between inpatient and outpatient care. The Blue App will help us collect data to make it easier to evaluate the work at the inpatient unit and will lay a foundation for future research in the field.

One important factor behind the development process was the support given to the project group from all levels in the organization, from stakeholders to staff [33]. Other crucial factors were related to the project group’s acceptance in the organization and to the committed team members whose perseverance has extended over several years. The organization benefits from the shared visions and team learning [33]. Finally, we want to highlight the importance of being offered enough time to work on this project.

### Limitations

The Blue App has been developed in a Swedish context, which limits its generalizability. Questionnaires are in Swedish, thereby excluding non-Swedish speaking patients. In the next version of The Blue App, the questionnaires will be available in the 4 most common languages at the unit (English, Dhari, Arabic, and French).

The design chosen for Study 2, in which selection is based on participation in Study 1, might affect inclusion and, therefore, could be regarded as a weakness. However, participation in Study 1 gives a thorough assessment of comorbidities at baseline, which is the strength of the design.

### Conclusions

A technically advanced and easy-to-use Web-based mobile phone app corresponding to the unit’s needs was developed, and 2 studies to evaluate its usefulness are planned.

## Abbreviations

IVR: interactive voice response
MADRS-S: Montgomery-Åsberg Depression Scale
MINI-KID: Mini International Neuropsychiatric Interview for Children and Adolescents
